# The Synergistic Effect of Combined Transforaminal and Caudal Epidural Steroid Injection in Recurrent Lumbar Disc Herniations

**DOI:** 10.7759/cureus.12538

**Published:** 2021-01-06

**Authors:** Sevket Evran, Ahmet Kayhan, Oguz Baran, Tahsin Saygi, Salim Katar, Enes Akkaya, Muhammet Arif Ozbek, Serdar Çevik

**Affiliations:** 1 Neurosurgery, Haseki Research and Training Hospital, Istanbul, TUR; 2 Neurosurgery, Koc University Hospital, Istanbul, TUR; 3 Neurosurgery, Medical Faculty of Balikesir University, Balikesir, TUR; 4 Neurosurgery, Sisli Etfal Hamidiye Research and Training Hospital, Istanbul, TUR; 5 Neurosurgery, Memorial Sisli Hospital, Istanbul, TUR; 6 School of Health Sciences, Gelisim University, Istanbul, TUR

**Keywords:** recurrent lumbar disc herniation, epidural steroid injection, transforaminal, caudal

## Abstract

Background

Recurrent lumbar disc herniation (RLDH) is one of the most common causes of chronic low back and leg pain. Although surgical treatment has high success rates in primary lumbar disc herniations, recurrence is not an uncommon clinic condition after the surgery. Considering the recurrent surgeries have lower success rates and higher risks, such as dural tear and nerve injury, alternative treatment modalities are needed for RLDH patients. Epidural steroid injections (ESI), particularly transforaminal steroid injection (TFESI) and caudal steroid injection (CESI), which are the alternative treatments to surgery, have not shown reasonable results in RLDH separately. In this study, we aimed to investigate the effects of combined TFESI and CESI (TFESI + CESI) treatment, which has been found successful in primary lumbar disc herniation (PLDH) and on pain control and quality of life in RLDH patients for the first time.

Materials and methods

A total of 71 patients, who had ESI treatment as only TFESI or TFESI + CESI because of RLDH in our clinic between March 2017 and February 2020, were investigated retrospectively. The visual analog scale (VAS) leg, VAS back, and Oswestry disability index (ODI) were used to assess leg pain, low back pain, and limitation of daily routine activities. Each assessment was done before the intervention and repeated at the third week, the third month, and the sixth month of injection, and the results were noted.

Results

Out of 71 patients, 38 were female and 33 male. Patients were divided into two subgroups according to the applied ESI methods as only TFESI (n = 32) and TFESI + CESI (n = 39). In the only TFESI group, the mean VAS leg score was 7.84, 4.63, 5.40, and 6.19 before, at the third week, the third month, and the sixth month of the injection, respectively. Also, in this group, the mean VAS back score was 8.06, 4.16, 4.88, and 5.97; the mean ODI score was 55.81, 34.31, 37.5, and 49.04 in the same respect. In the TFESI + CESI group, the mean VAS leg score was 8.20, 2.87, 3.64, 4.23; mean VAS back score 8.03, 3.05, 3.90, 4.08; mean ODI score 56.56, 28.05, 30.21, 33.64 before, at the third week, third month, and sixth month of the injection, respectively. The mean of the initial VAS leg, VAS back, and ODI scores was not found to be statistically significantly different between the two groups. The mean of all VAS leg, VAS back, and ODI scores was found to be lower in the TFESI + CESI group than the only TFESI group at each third-week, third-month, and sixth-month controls, and these differences were statistically significant. (p<0.0001 at each controls for VAS leg; p = 0.001 at third week, p = 0.002 at third month and p <0.0001 at sixth month for VAS back; p= 0.0003 at third week, p<0.0001 at third month, p<0.0001 at sixth month for ODI)

Conclusion

Our study demonstrates that TFESI + CESI treatment is an effective non-surgical treatment for RLDH. Considering the higher risks and lower success rates of recurrent surgeries, TFESI + CESI can be a potential treatment option for RLDH patients.

## Introduction

Recurrent lumbar disc herniation (RLDH) is one of the most common causes of failed back surgery syndrome (FBSS) [1]. Despite the high success rate of surgical treatment for primary lumbar disc herniations (PLDH), RLDHs are inevitable in 5%-15% of the cases [2].

The management of RLDH is challenging, and there is no certain data in the literature about choosing the most appropriate treatment option for these patients [3]. Additionally to epidural fibrosis-caused complications, such as dural tear and nerve injury, can be seen in RLDH, recurrent surgeries have lower success rates [4]. Considering the effectiveness of non-surgical treatment options in pain control, the usage of these modalities can lessen the incidence of FBSS and increase the comfort of both patient and surgeon.

Epidural steroid injections (ESI), which are among the minimally invasive treatment options and found more effective than conservative treatment in lumbar disc herniations [5], can be applied in three different ways: transforaminal (TFESI), caudal (CESI), and interlaminar (IESI). Steroids injected into the epidural area suppress the inflammation and ischemia, which are caused by the reaction of migrated leukocytes and various secreted neuropeptides, due to the occupation of the epidural area by the herniated nucleus pulposus, and alleviate the symptoms as a result [6].

Although the effectiveness of all three methods in PLDHs has been reported previously, there is a limited number of studies on RLDHs in the literature [7-9]. In these studies, IESI was found to be unsafe and has increased dural injury risk for patients with a history of lumbar spinal surgery [10]. Also, the lower efficiency of separately applied TFESI or CESI in RLDH than PLDH was reported [11-12].

It has been demonstrated that the combined application of ESIs as TFESI + CESI, which are known to be effective when applied separately in PLDHs, increases the effectiveness of the treatment [13]. Similarly, the combination of these techniques, which are not sufficient for the treatment of RLDHs separately, can be valuable in RLDHs. In this respect, we hypothesized that combining TFESI with CESI, which has anti-inflammatory and adhesiolysis effects around the nerve roots because it allows higher volume injections and can ensure synergistic effects for the treatment of RLDH patients. In this study, we aimed to investigate the effects of combined TFESI + CESI treatment in RLDH patients, which, to the best of our knowledge, have not been published previously in the literature.

## Materials and methods

A total of 71 patients who were referred to our clinic between March 2017 and February 2020 and were applied only TFESI or combined TFESI + CESI treatment due to RLDH were investigated retrospectively. All participants were informed in detail and their written informed consent forms were signed before the procedure.

Patient inclusion and exclusion criteria

**Figure 1:**
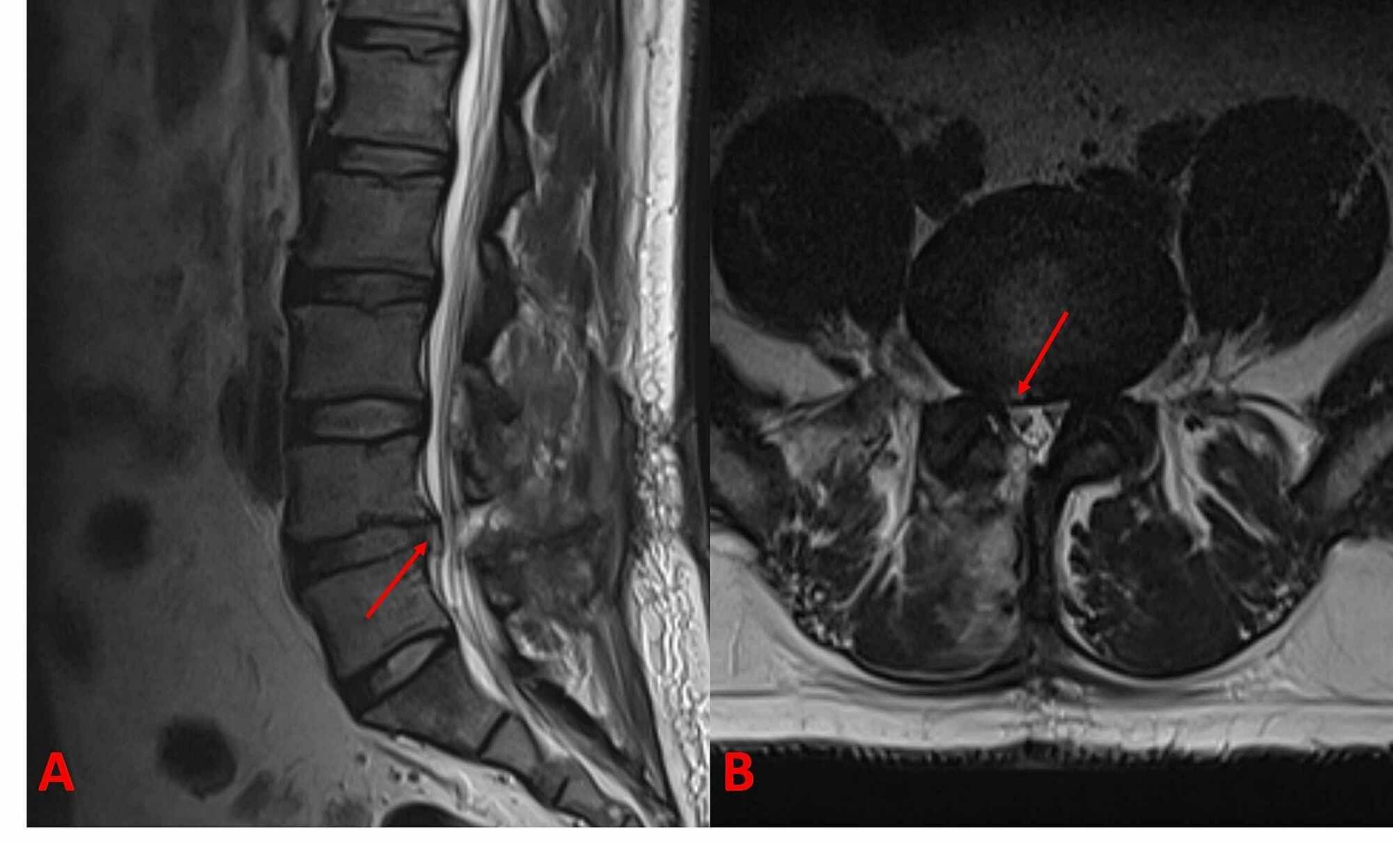
(A) Sagittal and (B) axial MRI sections of a patient with recurrent L4-5 disc herniation. Red arrows show the herniated fragment in each section. MRI: magnetic resonance imaging

Patients presented with low back and unilateral or bilateral leg pain who were found to have RLDH in lumbar MRI scans (Figure 1) and their radiological findings correlate with the complaints, did not have neurological deficits and whose symptoms did not improve with medical treatment (analgesic and myorelaxant drugs) or physiotherapy for more than six weeks, were included in the study.

Patients with lumbar degenerative spinal stenosis, spondylolisthesis, facet joint pain, adjacent segment degeneration, sacroiliac joint pain, discitis, pseudoarthrosis, arachnoiditis, segmental instability, spinal trauma, less than three months of complaints, partially benefited from medical treatment, infective or oncological disease, previous history of lumbar ESI treatment, severe metabolic or systemic disorders, required surgical treatment due to worsening neurological deficits and those who did not come to follow-ups were excluded from the study.

Intervention procedure

TFESI or TFESI + CESI procedures were performed only once for each patient by the same surgeon in the prone position. Blood pressure, electrocardiogram, pulse rate, and respiratory rate, oxygen saturation were monitored. Patients were not routinely sedated, and sedation with midazolam was used when necessary. After the procedures, patients were followed up for four to six hours in the recovery room to assess possible early complications. No major complications were observed. All patients were discharged within six hours after the procedure and hired for controls. Non-steroidal anti-inflammatory drugs were not prescribed to patients at discharge.

TFESI Procedure

**Figure 2:**
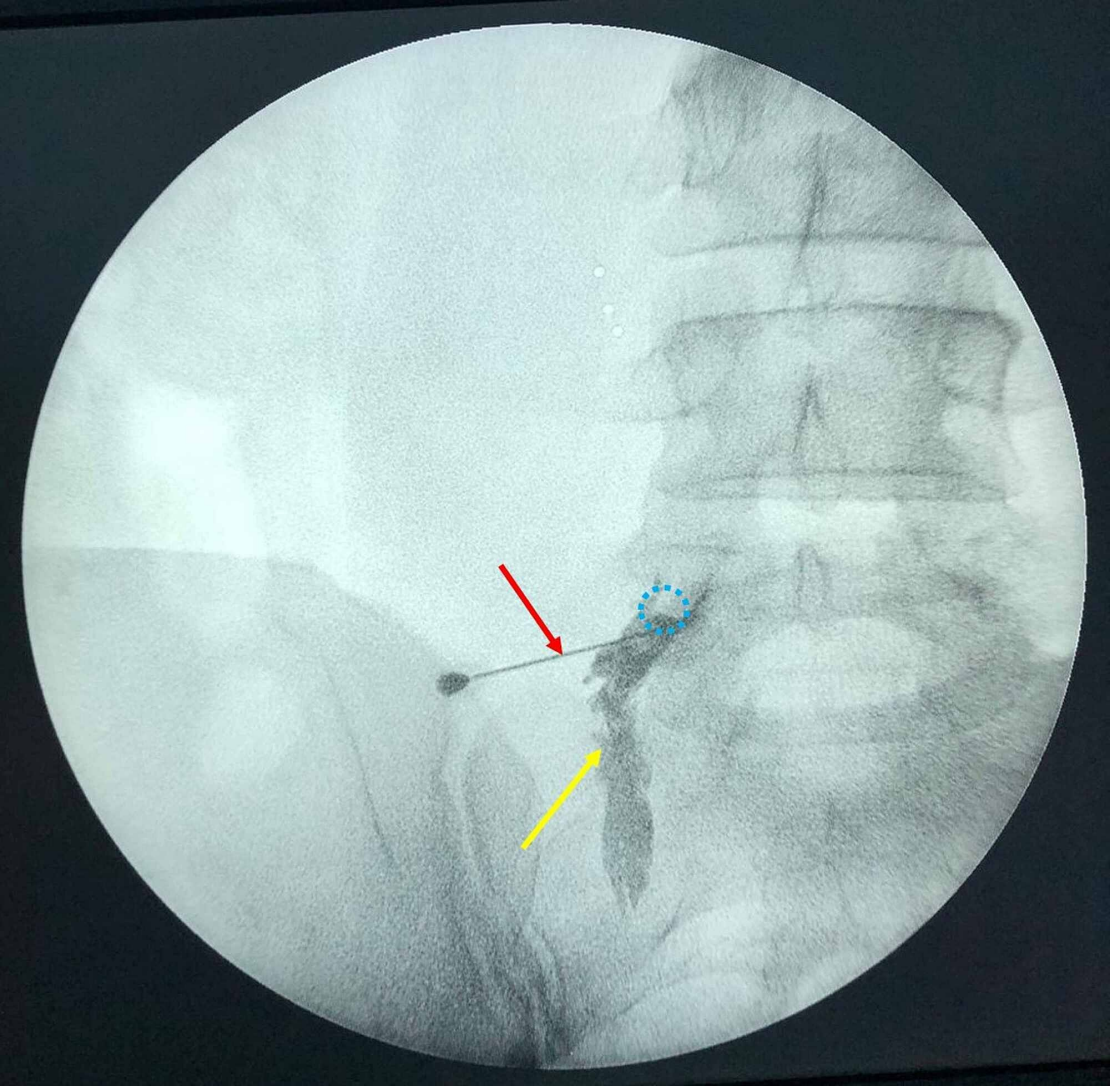
C-arm scope imaging of a patient with recurrent right L4-5 disc herniation during the TFESI procedure Spreading of the contrast agent (yellow arrow) from the tip of the needle (red arrow) to along the course of the right L5 nerve root, which arises from the right L5 foramen (blue dashed circle) is noted. TFESI: transforaminal epidural steroid injection

Following skin antisepsis and sterile dressing, the vertebra level to be injected was determined in the prone position with C-arm fluoroscopy in the A-P position. C-arm fluoroscopy was turned to a 15-degree oblique position and the intervertebral foramen was visualized. For local anesthesia, 1 mg 1% lidocaine was applied to the skin and subcutaneous tissue. The TFESI procedure was performed using the preganglionic approach with the technique described by Lee et al. [14]. Under the guidance of C-arm fluoroscopy, a 22-gauge, 90 mm spinal needle (Egemen International, Izmir, Turkey) was inserted toward the intervertebral foramen. Subsequently, C-arm fluoroscopy was placed in the A-P position and 1 ml of contrast agent (Omnipaque 300; iohexol, 300 mg iodine / mL, Amsterdam Health, Princeton, NJ) was injected to confirm the epidural spread (Figure 2). If the nerve root trace could not be seen, the needle was repositioned. After the location of the spinal needle was checked by C-arm fluoroscopy, aspiration was performed to check for bleeding and/or cerebrospinal fluid leakage. After checking for bleeding or cerebrospinal fluid leak, 40 mg (1 ml) methylprednisolone acetate (Depo-Medrol, Pfizer Pharmaceuticals Ltd, Luleburgaz, Kirklareli, Turkey) and 10 mg (2 ml) bupivacaine hydrochloride (Marcaine 0.5%, Astra Zeneca, Istanbul, Turkey) were injected in an average of two minutes.

CESI Procedure

**Figure 3:**
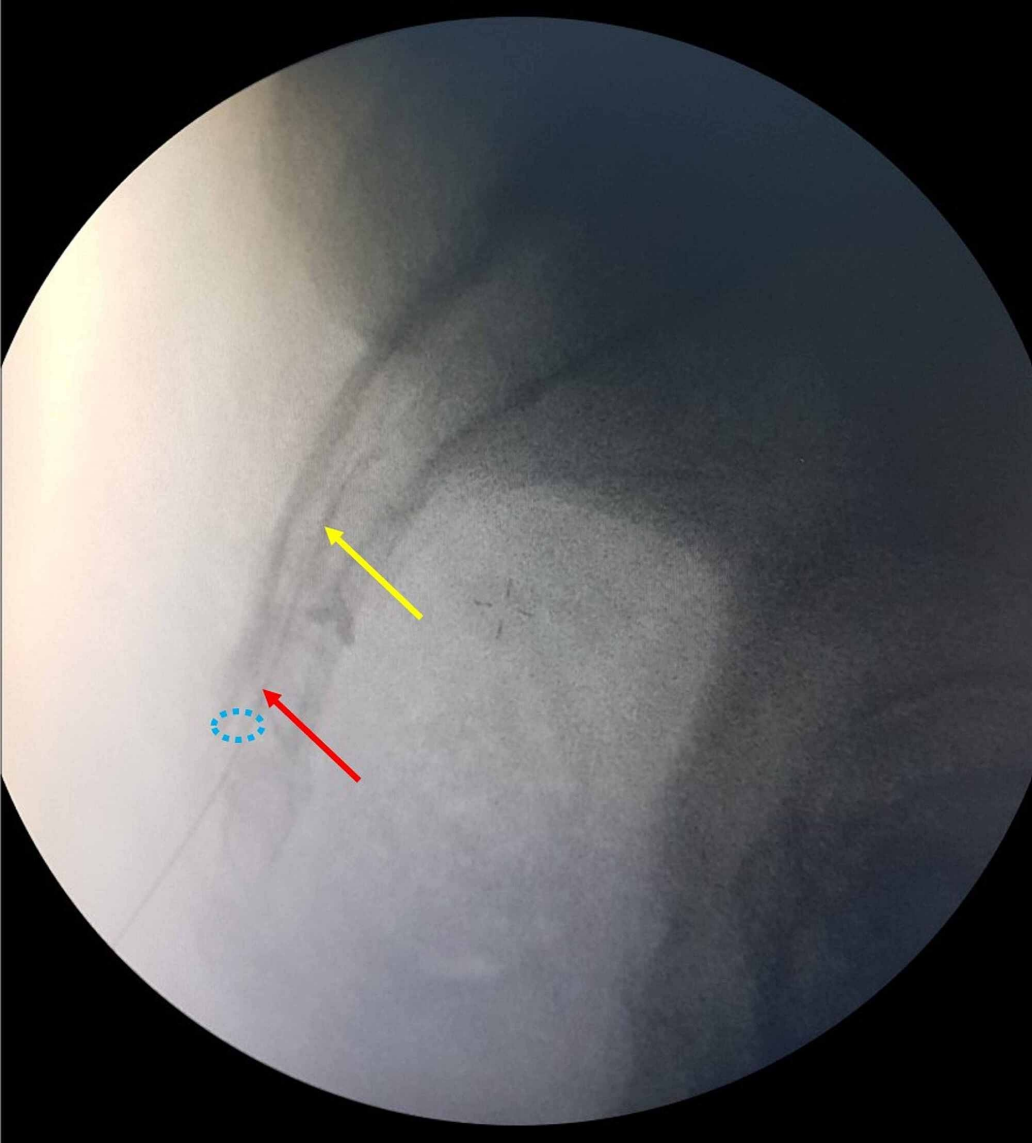
Lateral C-arm scope imaging during the CESI procedure Spreading of contrast agent (yellow arrow) from the tip of the needle (red arrow) into the epidural area and sacral hiatus (blue dashed circle) is noted. CESI: caudal epidural steroid injection

Following skin preparation and sterile dressing in the prone position on the operating table, sacral hiatus was identified and local anesthetic (1 mg, 1% lidocaine) was applied to the overlying skin and underlying ligaments. A 22-gauge, 90-mm spinal needle (Egemen International, Izmir, Turkey) was placed between the sacral cornua with a 45-degree angle under the C-arm scope control. After the control, the needle was directed more cranial, horizontal, and parallel to the ground then inserted through the sacrococcygeal ligament and entered into the caudal epidural area about 1-2 cm. After checking for bleeding or cerebrospinal fluid leakage by an aspiration test, the position of the spinal needle was confirmed by injecting 1 ml contrast agent (Omnipaque 300; iohexol, 300 mg iodine / mL, Amsterdam Health, Princeton, NJ, USA) (Figure 3) to control being in the epidural area. Forty (40) mg (1 ml) methylprednisolone acetate (Depo-Medrol, Pfizer Pharmaceuticals Ltd, Luleburgaz, Kirklareli, Turkey), 10 mg (2 ml) bupivacaine hydrochloride (Marcaine %0.5, Astra Zeneca, Istanbul, Turkey), and 17 cc 0.9% sodium chloride were injected slowly with a total of 20 cc.

Pre- and post-intervention assessment protocol

The leg and low back pain severity of patients were evaluated with the visual analog scale (VAS) score as VAS leg and VAS back separately. The participants scored their pain severity with values between 0 and 10 for this scale. Patients were asked to score 0 points for no pain and 10 points for the most intense pain of their life. 

The limitation of daily routine activities was assessed by the Oswestry Disability Index (ODI). ODI is a questionnaire that assesses the pain caused restriction with 10 items, including pain intensity, changing degree of pain, personal care, sleep quality, social life, and abilities of sitting, walking, standing, lifting, and traveling. Each subheading has six choices that correspond to scores ranging from zero to five according to the amount of disability. Scores are summed and then multiplied by two for evaluating over 100 points and higher scores point out more severe disability.

All patients were evaluated in the preoperative period and the postoperative third weeks, third months, and sixth months.

Statistical analysis

Descriptive statistics were used for continuous variables (mean, standard deviation, minimum, maximum, median). The paired t-test was used to compare the pre-injection and post-injection results of the TFESI + CESI group and the TFESI group average pain. A probability (p) value of <0.05 was considered statistically significant. All statistical analyses were performed using the IBM, SPSS Statistics version 22 (2013; IBM Corp., Armonk, NY).

## Results

A total of 85 patients were included in our study for a period of 35 months, but 14 patients were excluded from the study because they did not continue their follow-up. Thirty-eight (53.5%) of the remaining 71 patients were female and 33 (46.5%) were male. While 39 of 71 patients had both TFESI and CESI treatment in the same session, only TFESI was applied to 32 patients. Patients were divided into two groups according to the treatment they received. There was no statistically significant difference between the groups in terms of gender and affected disc level. The mean age of the only TFESI group was 49.7 (SD 12.46) and the TFESI + CESI group was 53.6 (SD 13.04) (p=0.194) (Table 1). There was no significant difference between the mean values of VAS/ODI scores and the level of herniated disc. The mean symptom duration was 5.17 ± 3.26 years in the TFESI + CESI group and 5.35 ± 3.07 years in the TFESI group (p = 0.798).

**Table 1:** Demographics and clinical data of the study population CESI: caudal epidural steroid injection; TFESI: transforaminal epidural steroid injection

n = 71		TFESI (n = 32)	TFESI + CESI (n = 39)
Age		49.7 ± 12.46	53.6 ± 13.04
Gender	Female (%)	18 (%25.35)	20 (28.17 %)
	Male (%)	14 (%19.72)	19 (26.76%)
Level	L2-L3 (%)	1 (%1.4)	2 (2.81%)
	L3-L4 (%)	3 (%4.23)	3 (4.23%)
	L4-L5 (%)	20 (%28.17)	23 (32.39%)
	L5-S1 (%)	9 (%12.68)	10 (14.09%)

Assessment after steroid injection

The VAS leg, VAS back, and ODI scores of patients at first application, third week, third month, and sixth month of the intervention are summarized in Table 2. 

**Table 2:** The mean values of VAS leg, VAS back, and ODI scores of groups, and their comparison CESI: caudal epidural steroid injection; TFESI: transforaminal epidural steroid injection; ODI: Oswestry Disability Index; VAS: visual analog scale

		TFESI	TFESI + CESI	p value
VAS leg	Pre-injection	7.84 ± 0.77	8.20 ± 1.19	= 0.128
	3rd week	4.63 ± 1.21	2.87 ± 1.15	< 0.0001
	3rd month	5.40 ± 1.32	3.64 ± 1.01	< 0.0001
	6th month	6.19 ± 1.06	4.23 ± 1.11	< 0.0001
VAS back	Pre-injection	8.06 ± 1.24	8.03 ± 1.14	= 0.897
	3rd week	4.16 ± 1.48	3.05 ± 1.17	= 0.001
	3rd month	4.88 ± 1.36	3.90 ± 1.19	= 0.002
	6th month	5.97 ± 1.15	4.08 ± 1.98	< 0.0001
ODI	Pre-injection	55.81 ± 8.04	56.56 ± 7.21	= 0.897
	3rd week	34.31 ± 7.86	28.05 ± 4.84	= 0.0003
	3rd month	37.5 ± 8.39	30.2 ± 5.14	< 0.0001
	6th month	49.04 ± 7.13	33.64 ± 5.52	< 0.0001

**Figure 4:**
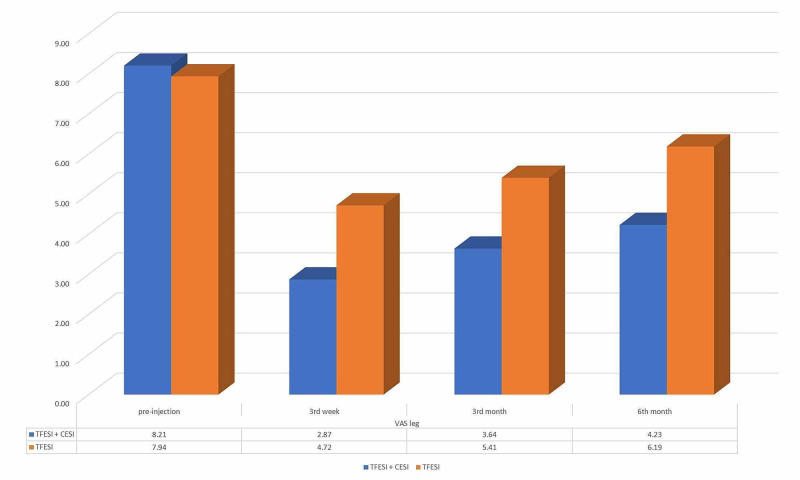
The mean VAS leg scores of TFESI + CESI and only TFESI groups at first appointment and 3rd week, 3rd month, 6th month of the injection. CESI: caudal epidural steroid injection; TFESI: transforaminal epidural steroid injection; VAS: visual analog scale.

The mean VAS leg score of patients who were applied only TFESI was 7.84 (SD 0.77, range 7-10) before the injection, 4.63 (SD 1.21, range 2-7) at the third week, 5.40 (SD 1.32, range 2-8) at the third month, and 6.19 (SD 1.06, range 4-8) at the sixth month of the injection. The mean of the VAS leg score was 8.20 (SD 1.19, range 6-10), 2.87 (SD 1.15, range 1-5), 3.64 (SD 1.01, range 2-6), and 4.23 (SD 1.11, range 2-6) at the first appointment, three weeks, three months, and six months after the injection respectively for patients who received combined TFESI + CESI. Although there was no statistically significant difference between the VAS leg scores of the TFESI and TFESI + CESI groups at the first appointment (p =.0128), the mean of the VAS leg score of TFESI + CESI patients was found to be statistically significantly lower than the only TFESI patients at the third week, third month, and sixth-month controls (p < 0.0001) (Figure 4).

**Figure 5:**
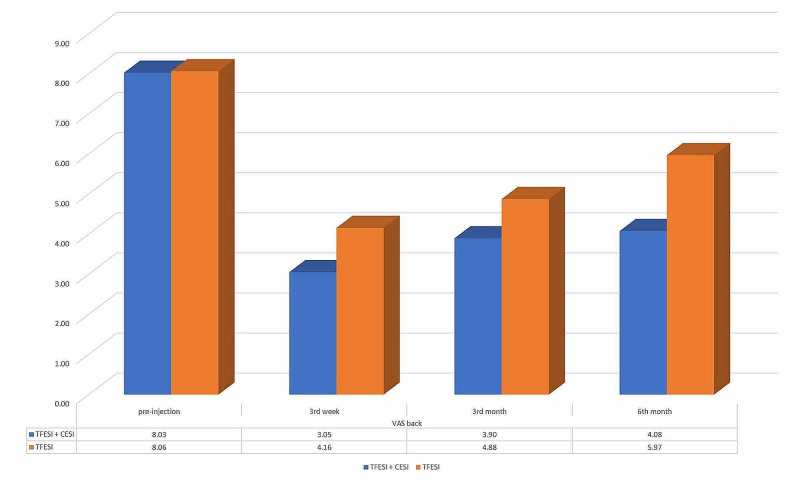
The mean VAS back scores of the TFESI + CESI and only TFESI groups at the first appointment and the third week, third month, and sixth month of the injection CESI: caudal epidural steroid injection; TFESI: transforaminal epidural steroid injection; VAS: visual analog scale

The mean VAS back score of only the TFESI group was 8.06 (SD 1.24, range 6-10) at the beginning, 4.16 (SD 1.48, range 1-7) at the third week, 4.88 (SD 1.36, range 2-7) at the third month, and 5.97 (SD 1.15, range 4-8) at the sixth month of the injection. In the TFESI + CESI group, the mean VAS back score was 8.03 (SD 1.14, range 5-10), 3.05 (SD 1.17, range 1-5), 3.90 (SD 1.19, range 1-5), and 4.08 (SD 1.98, range 2-8) in the same order. There was no statistically significant difference between the mean VAS back scores of the groups before the intervention (p=0.897), and the decrease of mean VAS back score after the injection was statistically significant in both groups. This decrease was more significant in the TFESI + CESI group at the controls (p = 0.001 for the third week, p = 0.002 for the third month, and p < 0.0001 for the sixth month) (Figure 5).

**Figure 6:**
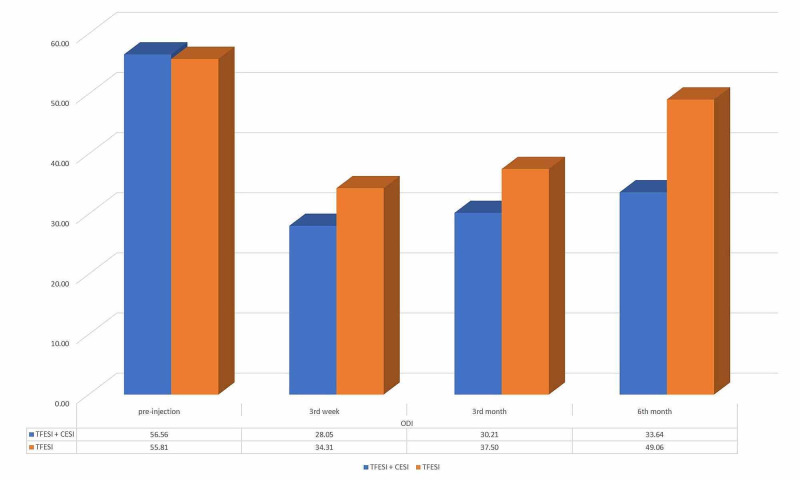
The mean ODI scores of the TFESI + CESI and only TFESI groups at the first appointment and third week, third month, and sixth month of the injection CESI: caudal epidural steroid injection; TFESI: transforaminal epidural steroid injection; ODI: Oswestry Disability Index

The initial mean ODI score of the TFESI group was 55.81 (SD 8.04) and the TFESI + CESI group was 56.56 (SD 7.21). The mean of the ODI score at the third week, third month, and sixth month of the injection was 34.31 (SD 7.86), 37.5 (SD 8.39), and 49.04 (SD 7.13) for the TFESI group and 28.05 (SD 4.84), 30.21 (SD 5.14), and 33.64 (SD 5.52) for the TFESI + CESI group, respectively. There was no statistically significant difference between the mean pre-interventional ODI scores of the groups (p = 0.897). Although the decrease of the mean ODI score as compared to the initial score was seen at all the third-week (p = 0.0003), third-month (p < 0.0001), and sixth-month (p < 0.0001) controls in both groups, the TFESI + CESI group was found to be with lower mean ODI scores at each control than the only TFESI group (Figure 6).

## Discussion

The persistent pain after discectomy is contributed by recurrent disc herniation, epidural fibrosis, spinal canal stenosis, laminectomy caused instability, and myofascial pain syndromes [15]. Also, recurring surgeries have a higher risk of nerve root injury and dural tear due to the presence of adhesions [4]. As a consequence, these risks cause less favorable outcomes and alternative treatment options may be beneficial for recurrent disc herniations.

ESI, one of these treatment options, can be applied by various methods, which especially include TFESI and CESI. Steroids injected into the epidural space reduce intraneural edema and venous congestion and thereby alleviate the ischemia and pain by direct and indirect actions [6]. The superiority of these methods to each other has been discussed widely in the current literature particularly for PLDH but knowledge about RLDH is restricted.

There are many publications indicating that TFESI is the most effective and preferred ESI method for PLDHs in the current literature [7-8,16]. This effectiveness of TFESI on pain control can be attributed to its various properties such as applicability to the compressed area directly, anti-inflammatory effects, inhibition of prostaglandin synthesis, dilution of inflammatory mediators, stabilization of cell membrane, suppressing immune-response, the increment of neuronal blood flow, and blockage on nociceptive C fiber conduction [7]. TFESI also has various superiorities to the CESI and IESI due to it allows an injection to the anterior side of the involved root [17] and requires a lower amount of drugs [18].

One of the other types of ESIs is CESI, which has less dural injury risk especially in patients with previous lumbar surgery history, and enables reaching the epidural area easier than both TFESI and IESI. Also, CESI has been found to be safer than IESI and TFESI for patients with bleeding diathesis caused by anticoagulant usage or coagulopathy [19]. CESI comes into prominence with its adhesiolysis characterized by the physical displacement of the nerves and the lysis of neuronal adhesions, which is caused by higher volume injections, particularly RLDH or FBSS [19-21]. Higher volume injections may lead to more dilution and the washing out of inflammatory mediators in the epidural area or more adhesiolysis achieved by higher hydrostatic pressure between nerve root sheet and adjacent tissues.

In CESI, steroids are administered through the caudal hiatus and maybe spread into areas beyond the desired target [22]. Also, the intravascular accidental injection of drugs during CESI has been reported due to the proximity of the venous plexus to the nerve roots in the caudal epidural area [23]. As a result, the presence of these conditions may decrease the effectiveness of treatment. Combining CESI with TFESI, which is a more target selective treatment, may help overcome cases in which CESI could not have been efficient alone.

One of the important points for ESI treatments is the effective duration of injections. Although TFESI had a longer effective duration than CESI [24], while CESI reached its maximum effect at two weeks after injection, this period was found to be six weeks for TFESI [16]. In this respect, the combination of these two types of injections can ensure a wider treatment-effective span.

Hypertrophied structures in the spinal canal block the effective distribution of steroids to the anterior epidural area in both TFESI and CESI [25-26]. In a similar way, a herniated disc fragment compresses the neural structures anteriorly and causes relative spinal stenosis, which can lead to the ineffective spread of injected agents in RLDH. Adhesions in RLDH patients can cause the malposition of the needle and insufficient distribution of injected drugs to the targeted area [27] in the TFESI procedure, and cause failure in pain relief but CESI has better results due to it allowing the injection of steroids in higher volumes [20-21]. Considering the coexistence of recurrent lumbar disc herniations and spinal stenosis, combining TFESI treatment with CESI seems a rational option.

There are many publications that investigate the efficiency of ESIs for PLDHS, but knowledge of RLDHs is limited in current literature. Karamouzian et al. compared the results of TFESI and CESI in RLDHs [11]. They suggested that TFESI had better results than CESI in RLDH patients but the difference was not statistically significant. When they compared the results with studies investigating the efficacy of TFESI and CESI in PLDH cases, both of these techniques were found to be less favorable in RLDH patients, and they indicated that the failure of TFESI treatment could be caused by adhesion and fibrosis. Also, Klessinger et al. reported that TFESI has less efficiency in patients with persistent radicular pain after microdiscectomy, particularly in RLDH [12]. In this respect, more favorable outcomes in our RLDH patients who had combined TFESI + CESI treatment may be caused by strengthening the reduced target selective effects of TFESI due to adhesions, with CESI's previously reported adhesiolysis influences.

Though there were no studies to the best of our knowledge for combined ESI treatment in RLDHs, there are limited studies that focused on the combined ESI treatment for PLDHs in current literature. One of these is Kircelli’s publication that compared the results of patients who had been treated by TFESI + CESI and TFESI alone. Additionally, they reported that combined treatment had better outcomes with increased quality of life and pain control in short- and long-term periods [13]. The underlying mechanism can be attributed to CESI’s disruption of adhesions, irrigation of the epidural space, and reducing inflammatory mediators' effects. Similarly, Hwang et al. reported TFESI combined with CESI treatment had better results than only TFESI treatment [28]. Although these studies demonstrate better results of combined ESI treatment in PLDH patients, our current issue is the first study of combined TFESI + CESI treatment efficiency for RLDH patients in literature with similar results as seen in PLDH patients.

Percutaneous adhesiolysis, which is a technique used for gaining epidural scarring lysis in patients with the lumbar post-surgery syndrome, caused permanent leg and low back pain. This technique is based on the usage of local anesthetics, steroids, hypertonic sodium chloride solutions, and hyaluronidases. Because the separate application of ESI methods was not found to be sufficient for lumbar post-surgery syndromes, including recurrent lumbar disc herniations, percutaneous adhesiolytics have come to the fore in this area and these agents have been found to be more effective than the separate application of ESIs particularly CESI [29]. Although the effects of combined ESI treatment in PLDH were demonstrated previously, it was observed that no researches focused on combining ESI methods that have easier application and fewer side effects in RLDH. We found that the combined CESI + TFESI application had synergistic effects and statistically significant clinical improvement in pain and functional capacity when compared to patients who received TFESI alone for RLDHs. In this respect, the combination of ESI procedures for RLDH’s treatment can be effective and triable. Studies comparing the effects of percutaneous adhesiolysis with combined CESI + TFESI in RLDHs, which have difficulties in their management, will enlight this issue in the future.

There are some limitations to this study. First of all, the study was carried out retrospectively. Also, only one type of TFESI and CESI approach was applied to patients, variants of the TFESI and CESI application methods were not considered, and their individual results could not be evaluated. Further studies can be planned regarding these points, for gaining wider knowledge.

## Conclusions

Considering the decreased effectiveness and increased risks of surgery, alternative treatment options are necessary for RLDHs. ESIs are frequently used and non-surgical treatment modalities in current clinical practice for lumbar disc herniations. The combined usage of ESI methods has become a current issue with higher success rates in PLDH but, to our knowledge, there were no studies that investigate their efficiency in RLDH previously. Our study demonstrates that combined TFESI + CESI can be a beneficial treatment option for RLDH patients whose management is challenging. Before planning further invasive treatment methods, combined ESI application may be useful for RLDH patients.
